# Combination of carbon ion beam and gemcitabine causes irreparable DNA damage and death of radioresistant pancreatic cancer stem-like cells *in vitro* and *in vivo*

**DOI:** 10.18632/oncotarget.3584

**Published:** 2015-03-14

**Authors:** Sei Sai, Toshifumi Wakai, Guillaume Vares, Shigeru Yamada, Takehiko Kamijo, Tadashi Kamada, Toshiyuki Shirai

**Affiliations:** ^1^ Medical Physics Research Program, Research Center for Charged Particle Therapy, National Institute of Radiological Sciences, Chiba, Japan; ^2^ Division of Digestive and General Surgery, Niigata University Graduate School of Medical and Dental Sciences, Niigata, Japan; ^3^ Radiation Risk Reduction Research Program, Research Center for Radiation Protection, National Institute of Radiological Sciences, Chiba, Japan; ^4^ Research Center Hospital for Charged Particle Therapy, National Institute of Radiological Sciences, Chiba, Japan; ^5^ Research Institute for Clinical Oncology, Saitama Cancer Center, Ina, Saitama, Japan

**Keywords:** carbon-ion beam, pancreatic cancer stem cell, gemcitabine, DNA repair

## Abstract

We try to elucidate whether a carbon ion beam alone or in combination with gemcitabine has advantages over X-ray in targeting putative pancreatic cancer stem-like cells (CSCs) *in vitro* and *in vivo*. Colony, spheroid formation and tumorigenicity assays confirmed that CD44+/ESA+ cells sorted from PANC1 and PK45 cells have more CSC properties than CD44−/ESA− cells. The number of colonies and spheroids formed from CSCs after carbon ion beam irradiation was significantly reduced compared to after X-ray irradiation, and they were extremely highly suppressed when carbon ion beam combined with gemcitabine. The relative biological effectiveness (RBE) values for the carbon ion beam relative to X-ray at the D10 levels for CSCs were 2.23-2.66. Expressions of multiple cell death-related genes were remarkably highly induced, and *large* numbers of γH2AX foci in CSCs were formed after carbon ion beam combined with gemcitabine. The highly expressed CSC markers were significantly inhibited after 30 Gy of carbon ion beam and almost lost after 25 Gy carbon ion beam combined with 50 mg/kg gemcitabine. In conclusion, a carbon ion beam combined with gemcitabine has superior potential to kill pancreatic CSCs via irreparable clustered DSB compared to a carbon ion alone or X-rays combined with gemcitabine.

## INTRODUCTION

Pancreatic ductal adenocarcinoma (PDAC), which constitutes more than 90% of pancreatic cancers in humans, is the fourth most frequent cause of cancer-related death world-wide [1, 2] and is characterized by a high rate of metastasis with high resistance to chemo-radiotherapy [3, 4]. In spite of great efforts to improve medical and surgical care over the past decades, little substantial progress has been made towards improving the PDAC prognosis, with the average overall 5-year survival rate still less than 5% [5, 6]. It has been reported that chemotherapy combined with conventional radiotherapy for locally advanced pancreatic cancer achieved about 17-25% of 2 year overall survival [7-9]. However, even in patients whose tumors initially are arrested or regressed the tumor still regrows after treatment. Resistance to chemo-radiotherapy is a major cause of treatment failure in pancreatic cancer. Therefore, there is a strong need for new therapeutic strategies targeting PDAC's chemo-radioresistant cells to elevate overall survival.

With increasing evidence supporting the existence of cancer stem-like cells (CSCs), pancreatic CSC populations have recently been identified based on cell membrane marker CD44^+^/ESA^+^ /CD24^+^ cells and CD133^+^ cells [10, 11]. CSCs represent a subpopulation of cells distinguishable from the bulk of the tumor based on their exclusive ability to drive tumorigenesis and metastasis [12-15]. CSCs are also considered responsible for therapy resistance and disease recurrence [16-18], and therefore represent interesting targets for new and more effective treatment strategies [19-22]. Thus, the development of new potent CSCs targeting therapeutics is highly desirable.

The heavy ion medical accelerator in Chiba (HIMAC) at the National Institute of Radiologic Science (NIRS) has treated more than 9000 patients with a variety of radioresistant tumors such as chordoma, sarcoma, and malignant melanoma, and has achieved promising results to date [23-30]. Heavy ion radiotherapy has been spotlighted not only in superior dose convergence but also the high biological effectiveness and is one of the minimally invasive treatments with the best quality of life (QOL), because heavy ion irradiation like carbon ion beams has several advantages compared to conventional photon therapy, such as cell-cycle and oxygenation-independence, and irreparable complex DNA damage. This is because the heavy ion beams have a well-defined range and insignificant scatter in tissues with well-localized energy deposition at the end of the beam path, called the “spread out bragg peak (SOBP)”, a unique physical characteristic of charged particle beams, and release enormous energy at the end of their range [31-33]. A phase I study to evaluate treatment of patients with locally advanced pancreatic cancer by carbon ion radiotherapy has been reported [34], and we have also achieved promising results for preoperative PDACs by carbon ion beam radiotherapy [35]. However, limitation of dose elevation because of important organs very nearby pancreas is one of most critical problem for carbon ion beam radiotherapy. Therefore, we speculated that a carbon ion beam combined with chemotherapy might allow the doses of irradiation to be reduced while still having some advantagein destroying PDAC. Several studies have reported that carbon ion beam combined with chemotherapy showed a small radiosensitizing effect, but this depends on the cell type and drugs (gemcitabine, cisplatin, camptonthecin) [36, 37]. Recently, our clinical trial showed that 58% of 2-year local control and 54% of 2-year overall survival rates without significant side effects were obtained by 45.6-55.2 GyE carbon ion radiotherapy combined with 1000 mg/m^2^ gemcitabine [38]. Based on the above reports in connection with our recent new finding that a carbon ion beam has a marked effect on colon as well as pancreatic CSCs, which are resistant to photon beams [39, 40], in the present study, we try to examine the effects of carbon ion beam alone or in combination with gemcitabine on putative pancreatic CSC survival, DNA repair, and xenograft tumor control compared to X-ray irradiation. To the best of our knowledge, this is the first study to explore whether a carbon ion beam combined with gemcitabine has a superior effect on pancreatic CSCs at relatively low doses compared to carbon ion beam alone or conventional X-ray irradiation *in vitro* and in vivo.

## RESULTS

### Determination of cancer stem-like cell properties of CD44+/ESA+ cells sorted from PNAC1 and PK45

**Figure 1:**
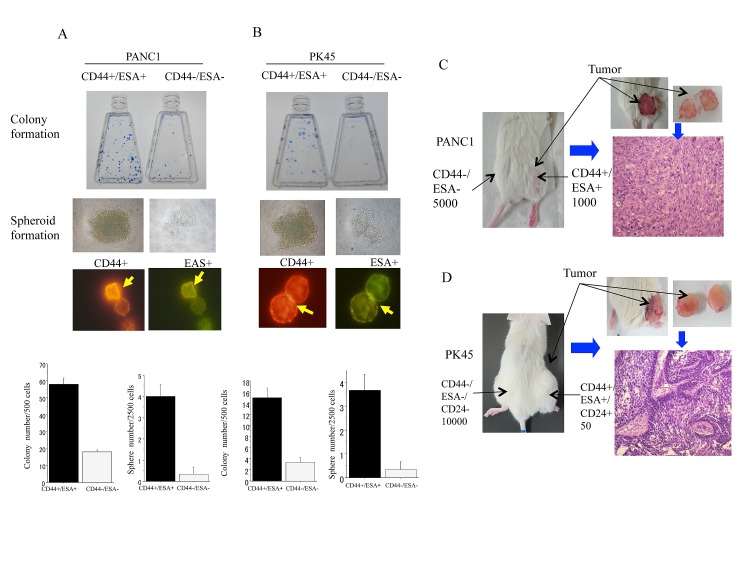
Colony, spheroid formation, and tumorgenicity of CSC and non-CSCs Colony and spheroid formation of CD44+/ESA+ cells and CD44−/ESA− cells delivered from PANC1 (A) and PK45 (B) cells after being in culture for 1-2 week. The number of colonies and the size of spheroids formed from CD44+/ESA+ cells are significantly higher than those of CD44−/ESA− cells. Representative photos of positive cancer stem-like cells are also displayed. *, p<0.01, compared to colonies or spheres formed from CD44−/ESA− cells. *In vivo* tumor formation ability of CD44+/ESA+ cells is significantly higher than CD44−/ESA− cells in NOD-SCID mice which delivered from PANC1 (C) and PK45 (D) cells.

When equal numbers of 500 cells were plated in a dish, CD44+/ESA+, cells from PNAC1, PK45 formed 55+ 3, 12 + 2 clones, whereas CD44+/ESA+ cells formed only 19 + 3, 2 + 1 clones (p<0.01). These data showed that CD44+/ESA+ pancreatic cancer cells had much greater clonal formation capacities than those of CD44−/ESA− cells (Figure 1A, B). After being in culture in 96-well round-bottomed spheroid plates (Sumilon, Sumitomo Bakelite Co., Tokyo. Japan) for 1 week, CD44+/ESA+ formed spheroid bodies (Figure 1A, B). The ability to form spheroid bodies in CD44+/ESA+ cells was significantly higher than in CD44−/ESA−S (p<0.01). Aliquots of 500 CD44+/ESA+ cells isolated from PK45, PNAC1 were transplanted subcutaneously into the right lower thigh of immunodeficient SCID mice and 5 × 10^3^ CD44−/ESA− cells were transplanted subcutaneously into the left lower thigh (Figure 1C). As shown in Table 1, only 50 cells of triple positive CD44+*/ESA+*/CD24+ cells could form a tumor whereas 1 × 10^4^ CD44−/*ESA*−/CD24− cells could not (Figure 1C). Collectively, our data suggested that CD44+*/ESA+*/CD24+, CD44+/ESA+ cells isolated from PK45, PNAC1 cells present the characteristics of CSCs. Because we need a much greater number of CSC cells with the sorting system for survival colony analysis and spheroid as well as γH2AX immunofluorescence analysis, we mainly used CD44+/ESA+ cells as CSCs in this experiment. We also confirmed that both CD44−/ESA− cells are non-CSCs, and based on the same reasons as mentioned above, we selected CD44−/ESA− cells as non-CSC in the present analyses. We also confirmed that CD44+/CD24+ cells sorted from MIA PaCa-2 and BxPc-3 have CSC properties [40].

**Table 1:** *In vivo* limiting dilution assays of sorted PANC1 and PK45 pancreatic cancer cells using surface markers (number of tumors formed/number of injections)

Groups	2 × 10^4^	1 × 10^4^	3 × 10^3^	5 × 10^2^	50
PANC1 Unsorted	3/5	1/6	0/6	-	-
CD44+/ESA+CD44+/ESA+/CD24+		5/5	4/5	3/5 3/3	1/5 1/3
CD44−/ESA−CD44−/ESA−/CD24-	2/5	1/4 0/4	0/4	-	-
p					
PK45 Unsorted	3/5	1/5	0/5	-	-
CD44+/ESA+CD44+/ESA+/CD24+		5/5	4/5	3/5	2/5 2/3
CD44−/ESA−CD44−/ESA−/CD24-		0/4 0/3	0/5	-	-
p		<0.01	<0.01	<0.01	<0.01

*p*<0.01 compared with results from marker-negative cells.

### Changes in proportion of CD44+/ESA+ cells after carbon-ion beam alone or in combination with gemcitabine

**Figure 2:**
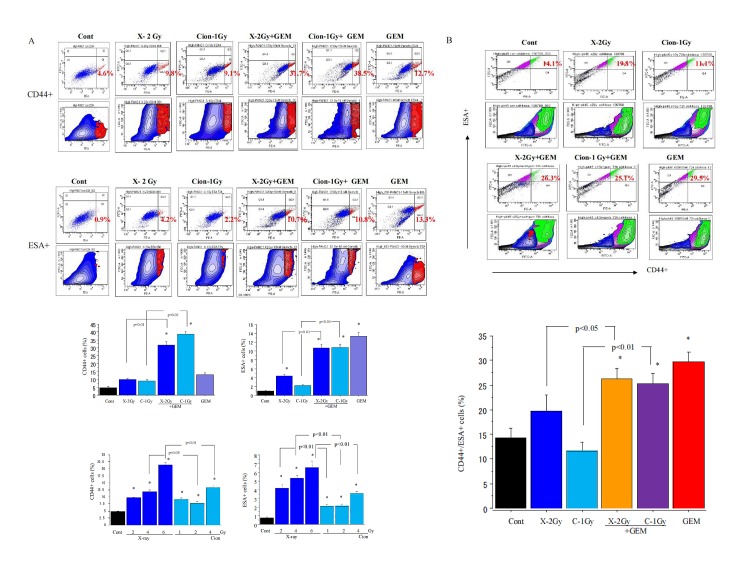
(A) Percentage changes of CD44+, ESA+ cells by FACS analysis 96 h after carbon ion beam, X-ray, 10 nM gemcitabine (GEM) alone or in combination with 10 nM gemcitabine in PANC1 cells. (B) Percentage changes of double CD44+/ESA+ cells by FACS analysis 72h after carbon ion beam, X-ray10 nM gemcitabine alone or in combination with 10 nM gemcitabine in PK45 cells.

In PK45, PNAC1 cells, changes in the percentages of CD44+, ESA+ cells 72 h or 96 h after X-ray or carbon ion irradiation were investigated. The percentage of CD44+, ESA+ cells in unirradiated PANC1 cells was about 4.7%, 0.9%, and it dose-dependently increased more than 2-5 fold after X-ray irradiation, but only around 2 folds by carbon ion beam alone at which the doses induced equivalent effects by X-ray (Figure 2A). However, the percentage of CD44+ and ESA+ cells was extremely highly increased by more than 5-10 fold when X-ray or carbon ion beam were combined with 10 nM of gemcitabine. Gemcitabine alone treatment also significantly increased the percentage of CD44+ and ESA+ cells (Figure 2A). The proportion of double positive CD44+/ESA+ cells in PK45 cells after X-ray, carbon ion beam alone or in combination with gemcitabine showed the same tendency (Figure 2B).

### Surviving fraction of unsorted PNAC1, PK45 cells and CD44+/ESA+, CD44−/ESA− cells sorted from PNAC1 and PK45 cells after carbon-ion beam or X-ray irradiation after carbon ion beam

**Figure 3:**
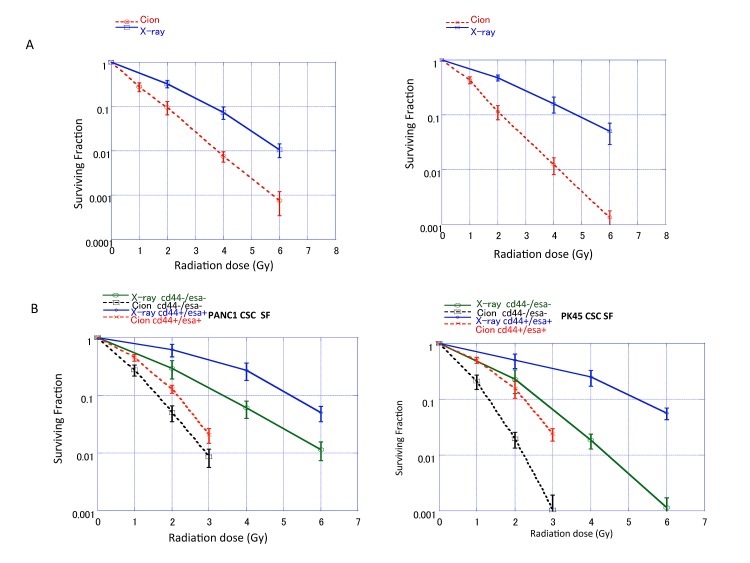

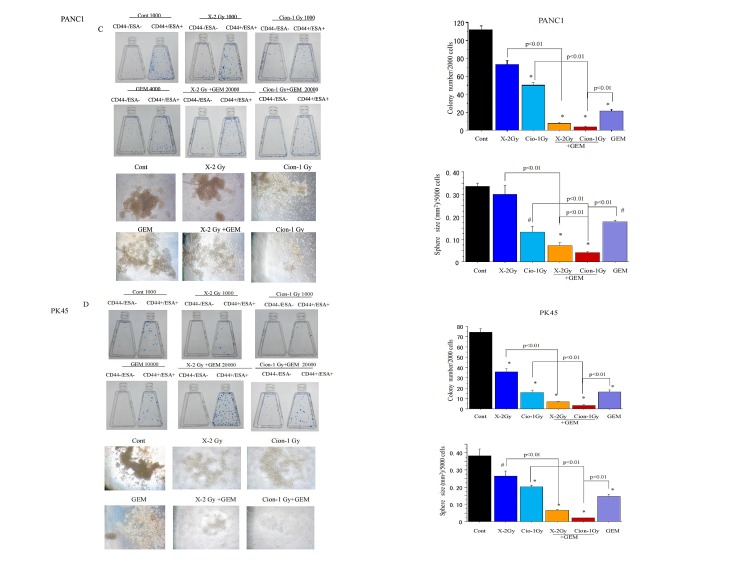
(A) Surviving fraction of unsorted PANC1 and PK45 cells. (B) Cancer stem-like CD44+/ESA+ cells and non cancer stem-like CD44−/ESA− cells delivered from PANC1 and PK45 cells plated immediately after carbon ion beam or X-ray irradiation. The graphs show the mean and standard error calculated from three independent experiments. (C) Representative photos and quantification of colony and spheroid formation of CSCs and non-CSCs delivered from PANC1 (C) and PK45 cells (D) after X-ray, carbon ion beam, 10 nM gemcitabine (GEM) alone or X-ray, carbon ion beam in combination with gemcitabine. The spheroid formation was observed 7 days after exposure of the plated cells to carbon ion beam or X-ray. Gemcitabine was added to the medium just 1-2 h before irradiation for 24 h. The graphs show the mean and standard error calculated from three independent experiments.

The surviving fractions for the unsorted PK45 and PANC1 irradiated with X-rays or carbon ion beams decreased exponentially with increasing doses. Based on these survival curves, the *RBE* values calculated by the *D10*, which is determined as the dose (Gy) required to reduce the surviving fraction to 10%, relative to X-rays, is about 1.85 to 2.10 for carbon-ion beams (Figure 3A). Based on these survival curves, the *RBE* values calculated at the *D10* level for CSCs were calculated to be about 2.43 to 2.48, whereas RBE values for non-CSCs were about 1.94. The results show that the surviving fractions for CD44+/ESA+ cells are significantly higher than CD44−/ESA− cells after irradiation with either X-rays or carbon ion beams (Figure 3B), suggesting that CSCs showed resistance to both X-rays and carbon ions. RBE values for unsorted and sorted CSCs and non-CSCs of carbon ion beams relative to X-rays are summarized in Table 2.

**Table 2:** RBE values at D10 level for unsorted PANC1 and PK45 cells and sorted cancer stem-like and non-cancer stem-like cells

Cells	X-ray	C-ion	RBE
PANC1 unsorted	3.62 ± 0.20 Gy	2.11± 0.10 Gy	1.71
PK45 unsorted	4.84 ± 0.12 Gy	2.20 ± 0.05 Gy	2.18
Cells	X-ray	C-ion	RBE
PANC1 CD44+/ESA+	5.13 ± 0.11Gy	2.12 ± 0.10 Gy	2.43
CD44−/ESA−	3.26 ± 0.13 Gy	1.72 ± 0.06 Gy	1.94
PK45 CD44+/ESA+	5.26 ± 0.13Gy	2.24 ± 0.10 Gy	2.35
CD44−/ESA−	2.73 ± 0.10 Gy	1.44 ± 0.07 Gy	1.93

### Colony and spheroid formation ability of CD44+/ESA+ cells sorted from PK45 and PNAC1 cells after carbon-ion beam or X-ray alone or in combination with gemcitabine

The number of colonies formed from CD44+/ESA+ cells was significantly decreased after X-ray, carbon ion beam, and gemcitabine-alone treatments, and it was further remarkably reduced when carbon ion beam was combined with gemcitabine (Figure 3C, 3D). The spheroid size of cancer stem like CD44+/ESA+ cells delivered from PANC1 (Figure 3C) and PK45 cells (Figure 3D) was significantly reduced by carbon ion beam-alone, or gemcitabine-alone treatment but not by X-ray irradiation alone, and it was extremely heavily decreased after gemcitabine combined with either X-ray or carbon ion beam. However, small spheroids were still formed after X-ray combined with gemcitabine. In comparison, spheroid formation could not form when carbon ion beam combined with gemcitabine. No spheres were formed in non-CSCs with or without X-ray, carbon ion beam, gemcitabine alone or in combination treatment (data not shown).

### Expression changes of various genes after carbon-ion beam alone or in combination with gemcitabine by RT PCR Array analysis

**Figure 4:**
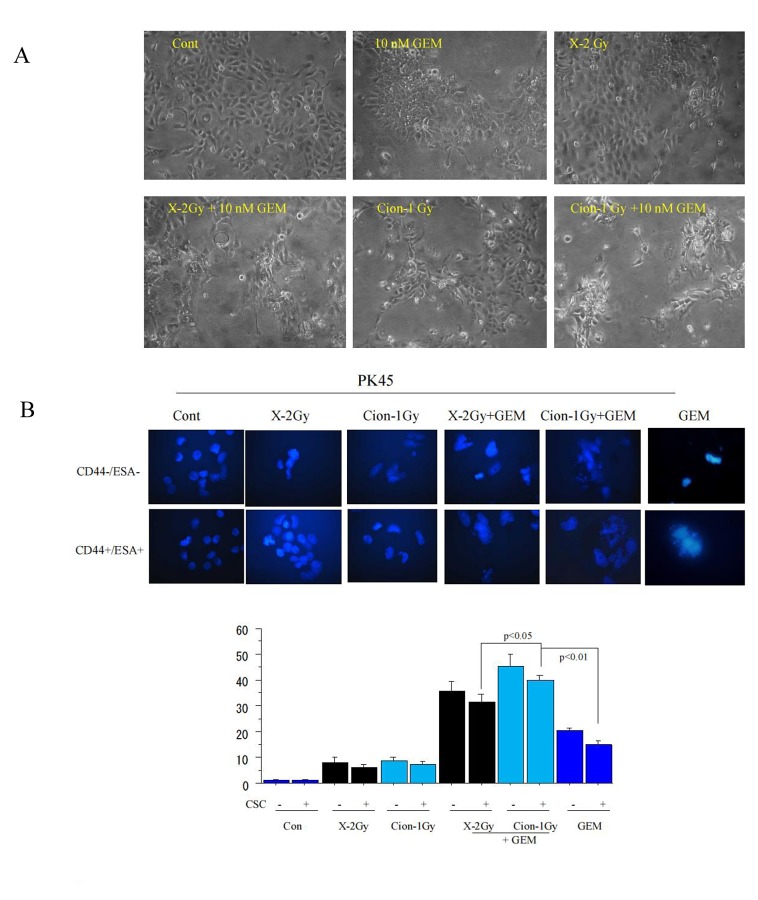

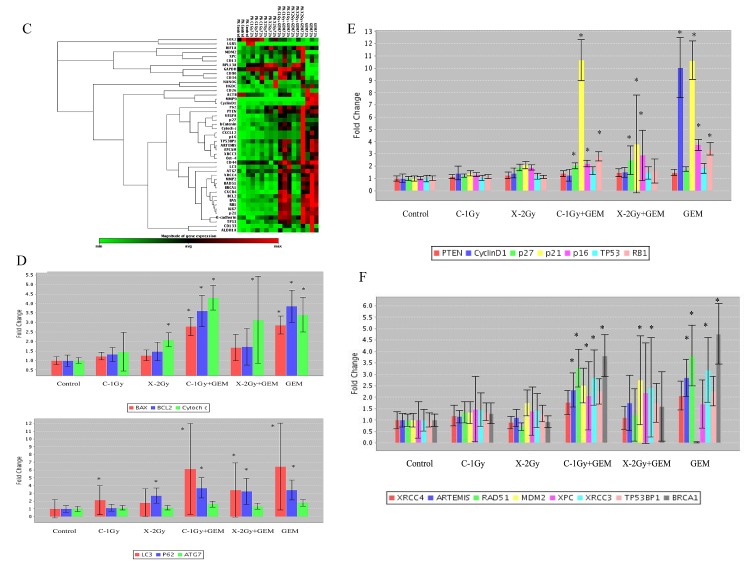

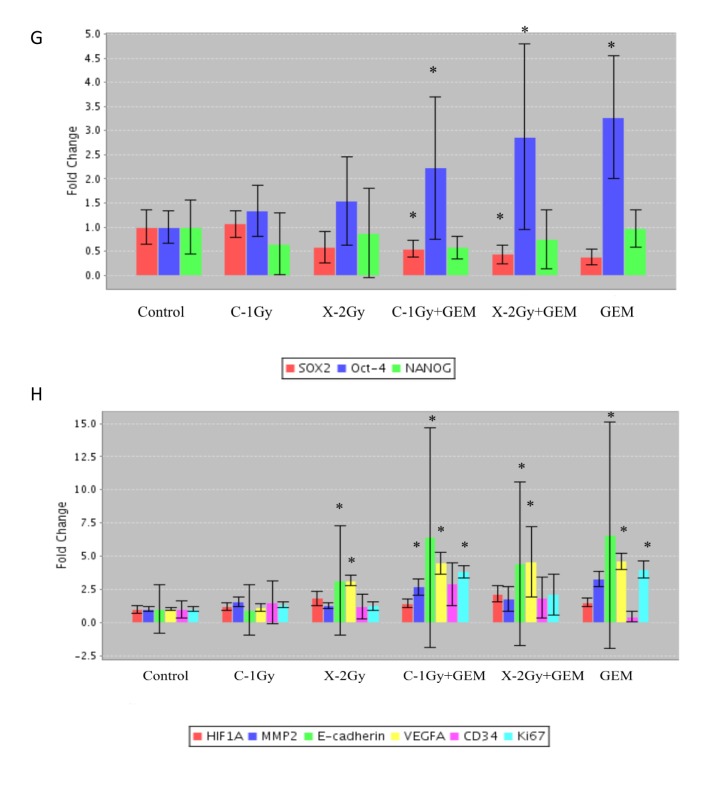
(A) Morphological changes of PK45 cells at 72 h after treatment with carbon ion beam alone, X-ray alone or in combination with gemcitabine (GEM). (B) Representative photos and quantification of apoptosis in CD44+/ESA+ cells and CD44−/ESA− cells 48 h after carbon ion beam alone, X-ray alone or in combination with gemcitabine (GEM) in PK45 cells. (C) Clustergram of Custom RT-PCR Array of PK45 cells 72 h after carbon ion beam alone, X-ray alone or in combination with 10 nM gemcitabine.9GEM) Expression changes of apoptosis and autophagy (D) senescence (or cell cycle) (E), DNA repair (F), stemness angiogenesis and metastasis-related genes after carbon ion beam alone, X-ray alone or in combination with gemcitabine 9GEM) in PK45 cells. *. *p* < 0.01 compared to control.

Morphological changes and apoptotic cells of PK45 stained with Hoechst 33342 after X-ray, carbon ion beam alone or in combination with gemcitabine are shown in Figure 4A, B. The apoptotic CSCs and non-CSCs were predominantly induced by carbon ion beam combined with gemcitabine compared to carbon ion, X-ray, gemcitabine alone or X-ray combined with gemcitabine. Figure 4C shows a clustergram of Custom RT^2^ Profiler PCR Array analysis after treatment with carbon ion beam in combination with gemcitabine for PK45 cells. Apoptosis-related gene expressions such as Bax, cytochrome c and Bcl2, as well as autophagy-related genes such as LC3, p62, but not ATG7, were significantly elevated by carbon ion beam combined with gemcitabine or gemcitabine alone compared to carbon ion beam, X-ray alone or X-ray combined with gemcitabine (Figure 4D). In addition, expression of senescence-related genes such as p21, p16 and p27 was remarkably increased by carbon ion beam combined with gemcitabine compared to carbon ion and X-ray alone or X-ray combined with gemcitabine (Figure 4E). Gemcitabine-alone treatment simultaneously remarkably increased the expression of cyclin D1, p21 and p16. Interestingly, expressions of DNA damage and repair-related genes such as ARTEMIS, Rad51, TP53BP1, BRAC1 were significantly enhanced after carbon ion beam combined with gemcitabine compared to carbon ion beam alone, X-ray alone or X-ray combined with gemcitabine (Figure 4F). Expressions of cancer stemness-related genes such as Sox-2, and Nanog-1 but not Oct-4, were showed decreasing trend by carbon ion beam combined with gemcitabine compared to that of carbon ion beam, X-ray alone or X-ray combined with gemcitabine (Figure 4G). However, expressions of angiogenesis-related genes such as HIF1α, VEGF and tumor invasion-related genes like MMP2, MMP9, E-cadherin and β-catenin were increased by either carbon ion beam or X-ray irradiation alone and/or in combination with gemcitabine compared to (Figure 4H).

### γH2AX foci formation in CD44+/ESA+ and CD44−/ESA− cells after carbon-ion beam alone or in combination with gemcitabine

**Figure 5:**
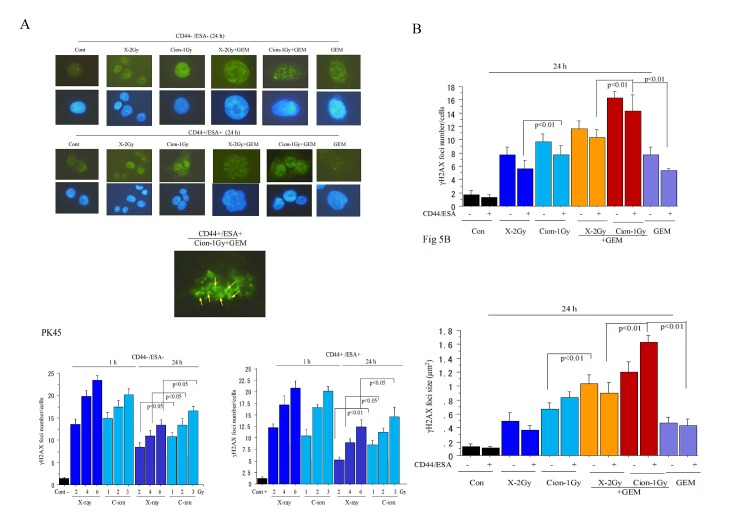
Representative photos (A) and quantification (B) of nuclear γ foci formation at 1 h, 24 h carbon ion beam, and X-ray alone or in combination with 10 nM gemcitabine (GEM) is presented according to radiation dose response. Data represent mean ± SD. ^*^p < 0.01 compared to non-CSCs. Quantification and representative photos of nuclear γH2AX foci lager than 1.5 μm^2^ after 24 h carbon ion irradiation in CD44+/ESA+ cells delivered from PK45 cells also displayed (C). Arrows indicate γH2AX foci lager than 1.5 μm^2^. ^*^p < 0.01 compared to γH2AX foci sizes in X-ray irradiated cells.

A high number of γH2AX foci formed at 1 h after X-ray or carbon ion irradiation both in CD44+/ESA+ and CD44−/ESA− cells which had been sorted from PK45. However, at 24 h after carbon ion irradiation, the induced γH2AX foci level remained significantly higher than that of X-ray irradiated cells with isoeffective dosages (Figure 5A). Furthermore, not only a great increase in the number but also in the size of foci (clustered DSB) was frequently found in carbon ion beam combined with gemcitabine-treated cells (Figure 5A, B). Interestingly, the big-sized γH2AX foci were observed more frequently in CD44+/ESA+ cells than in CD44−/ESA−^−^ cells. In addition, the number of γH2AX foci formed in CD44+/ESA+ cells decreased more significantly than in CD44−/ESA− cells after X-ray irradiation (Figure 5A, B). The same results were also obtained in CD44+/ESA+ and CD44−/ESA− cells which had been sorted from PANC1 (data not shown).

### Tumor growth control by carbon-ion beam alone or in combination with gemcitabine

Transplanted PK45 xenograft tumors grow fast without any treatment and the tumor volume became more than 480 mm3 after being subcutaneously implanted in the mice for 1-month. Treatment with X-ray (30 Gy) effectively suppressed tumor growth and reduced the tumor size and volume by about 10%, but the tumor rapidly re-grew after 4 weeks and to double in volume after another month. In contrast, treatment with carbon-ion (30 Gy) radiation dramatically dereased tumor size and volume by a factor of 2 in the first week and then gradually decreased. The tumor was reduced to the same size before radiation after one month and actually became less than half in volume after 8 weeks, and finally disappeared after 12 weeks without any regrowth and relapse. To determine the possibility of tumor growth control by carbon-ion or X-ray in combination with gemcitabine, the xenograft tumors were also treated with carbon ion 25 Gy or X-ray 35 Gy combined with 50 mg/kg gemcitabine ip. Carbon-ion irradiation with 25 Gy in combination with gemcitabine can suppress tumor growth without re-growth after 7-8 weeks. As expected, treatment with 35 Gy X-ray in combination with gemcitabine failed to control tumor growth. Xenotransplanted tumor control possibility by carbon-ion and X-ray alone or in combination with gemcitabine at various doses is summarized in Table 3.

**Table 3:** Therapeutic efficacy of X-ray and carbon ion beam alone or in combination with gemcitabine in xenograft tumor from PK45 pancreatic cancer cells (16-week follow up)

Group	Mice (n)	Complete response	Partial response
Unirradiated GEM (50mg/kg × 4)	5 5	- 0	- 5
X-ray 15Gy 35Gy 35Gy+GEM (50mg/kg) 60Gy	5 5 10 5 5	0 0 3 1 2	5 5 7 4 3
Carbon-ion 15Gy 25Gy 25Gy+GEM (50mg/kg) 35Gy	5 5 5 5 5	0 1 5 5 5	5 4 0 0 0

Complete response: the tumor was completely controlled without regrowth during the observation period. Partial response: the tumor was not completely controlled and regrowth during the observation period.

### Histopathological changes after carbon ion beam alone or in combination with gemcitabine

**Figure 6:**
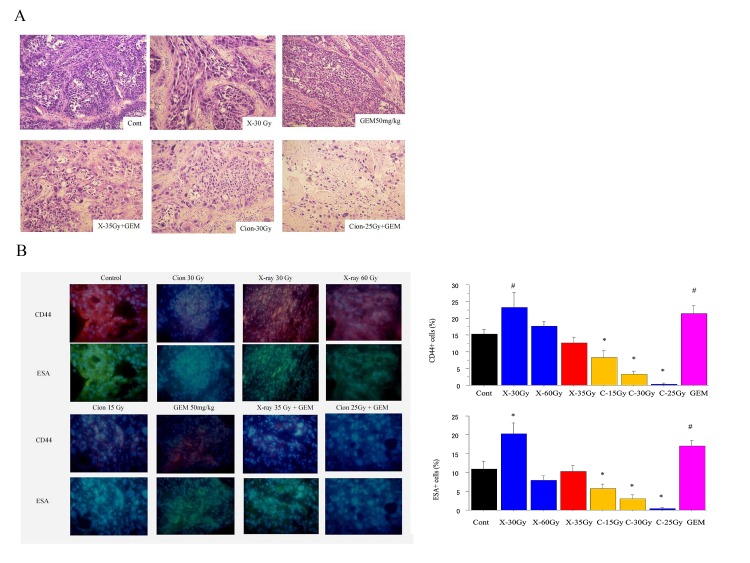
**A.** Histopathological changes 1 month after X-ray, carbon ion beam, gemcitabine (GEM) (50 mg/kg) alone and its combination in PK45 xenograft tumors. Gemcitabine was given alone once a week or 1 week prior to irradiation with X-ray or carbon ion beam for 4 times. **B.** Representative photos and quantification immunofluorescence staining of CD44 and ESA 1 month after X-ray, carbon ion beam, gemcitabine alone and in combination in PK4 xenograft tumors. #, *p*<0.05 compared to control; *. *p* < 0.01 compared to control.

Most of the tumor cells were not disrupted by 30 Gy X-rays or 15 Gy carbon ion irradiation, but were partially or predominantly destroyed by 25 Gy or 30 Gy of carbon ion beam with necrosis, cavitation and fibrosis. It is very clearly shown that most of the tumor cells were destroyed after being irradiated with a 35 Gy carbon-ion beam alone or a 25 Gy carbon ion beam combined with 50 mg/kg gemcitabine (Figure 6A).

### *In vivo* changes of expression of CD44 and ESA after carbon-ion beam alone or in combination with gemcitabine

Immunofluorescence analysis showed that 30 Gy of carbon-ion beam predominantly suppressed expression of both CD44 and ESA. In comparison, even 60Gy X-rays did not reduced expression of CD44 and ESA (Figure 6B). The CD44 and ESA expression was almost lost by 25 Gy of carbon-ion beam in combination with 50 mg/kg of gemcitabine, whereas these CSC markers were still expressed by 35 Gy of X-rays in combination with gemcitabine. This finding suggests that high doses of carbon ion beam alone or carbon ion beam with relatively lower doses combined with gemcitabine can more effectively destroy CSCs compared to that a high dose of X-rays, alone or combined with gemcitabine (Figure 6B).

## DISCUSSION

We found that *in vitro* RBE values for PNAC1 and PK45 cells after carbon ion beam at the center of SOBP (average LET is around 50-keV/μm) relative to the X-rays are about 1.71 to 2.18 when calculated by the D10 levels. RBE values are various dependent on LET as well as cell types, and our results from this study are almost in line with our previously reported other pancreatic cell lines [40] and also place within other reported literatures using 50-80 keV/μm carbon ion beams on several human cancer cells [42]. It has been demonstrated that various cancer stem cell markers exist according to the tumor types, and CD133 is not always expressed as a CSC marker [43]. In the present study, we did not detect CD133 in both PNAC1 and PK45 cells, this is the same as the case of MIA PaCa-2 and BxPc-3 cells. We found that CD44+/ESA+, CD44+/CD24+ cells have a significantly higher possibility for colony and tumor sphere formation than CD44−/ESA− and CD44^−^/CD24^−^ cells. The *in vivo* tumorigenicity study showed that the tumorigenicity of pancreatic cancer cells is much higher than CD44−/ESA− and CD44−/CD24− cells. As expected, triple positive CD44+/ESA+/CD24+ cells have superior CSC properties compared to those of double positive CD44+/ESA+ or CD44+/CD24+ cells.

In the present study, FACS analyses showed that the proportion of cancer stem-like CD44+/ESA+ cells was more highly enriched after X-rays compared to carbon ion irradiation. However, either X-ray or carbon ion beam combined with gemcitabine significantly increased CSC proportion. The percentages of cancer stem cell-like CD44+/ESA+ cells increased markedly by 5-10-fold after X-ray irradiation, whereas the proportion of these cells only doubled or decreased after carbon ion irradiation. It is suggested that cancer stem cell-like CD44+/ESA+ cells may be resistant to X-rays resulting in selective killing of non-cancer stem cell-like CD44−/ESA− cells, leading to an increase in the relative proportion of CSCs, whereas carbon ion irradiation may kill both cancer and non-CSCs at the same time, with relatively small changes in the proportion of CSCs in the population. This finding is consistent with our and other previous reports [39, 40]. To directly determine the radiosensitivity of pancreatic cancer stem-like CD44+/ESA+ cells for carbon ion beam, or X-ray irradiation, alone or in combination with gemcitabine, a colony assay was performed. Based on dose-response curves for cell-killing effect on CSCs and non-CSCs after irradiation with either X-rays or carbon ion beams, the CSCs showed resistance to both X-rays and carbon ions compared to non-CSCs. However, CSCs were significantly radiosensitized when carbon ion beam was combined with gemcitabine. In addition, spheroid formation abilities were predominantly reduced by carbon ion beam combined with gemcitabine compared to that of X-ray combined with gemcitabine.

The surviving fractions for the cancer stem like cells after irradiation with X-rays or carbon ions decreased exponentially with increasing doses. The *RBE* values calculated at the *D10* level for CSCs delivered from PK45, PNAC1 were about 2.23 to 2.66, suggesting that the carbon ion beam has a promising potential to destroy CSCs. In contrast, *RBE* values at the *D10* level for non-CSCs delivered from PK45, PNAC1 were only 1.94, implying that the difference in killing pancreatic cancer cells between carbon ion beam and X-ray irradiation might mainly result from the strong effects on CSCs. Taken together, these results may explain why the proportion of CSCs after irradiation with X-rays is more enriched than carbon ion beams.

It has been demonstrated that gemcitabine radiosensitizes pancreatic cancer cells accompanied with apoptosis and autophagy [36, 44-47]. Gemcitabine has also been shown to induce senescence in pancreatic cancer cells [48]. In the present study, we firstly found that after treatment with carbon ion beam in combination with gemcitabine for PK45 cells, not only apoptosis-related gene expressions such as Bax, cytochrome c and Bcl2 but also autophagy-related genes such as LC3, p62 as well as senescence-related genes such as p21, p16 were significantly elevated compared to carbon ion beam alone, X-ray alone or X-ray combined with gemcitabine, suggesting that carbon ion beam combined with chemotherapy may have more power to induce multiple cell death. Gemcitabine alone also induced multi cell death related gene expressions, but simultaneously significantly enhanced expression of cyclin D1 oncogene. Embryonic stem cell factors have shown to be closely related with cancer stemness and also associated with radioresistance [49, 50]. Interestingly, strong downregulation of expressions of the reprogramming factors such as Sox-2, Nanog-1 but not Oct-4 were found after carbon ion beam combined with gemcitabine compared to that of carbon ion and X-ray alone or X-ray combined with gemcitabine, indicating that carbon ion beam combined with chemotherapy may have more potential to regress pancreatic cancer cell stemness. Expressions of DNA damage and repair-related genes such as ARTEMIS, Rad51, TP53BP1, BRAC1 were significantly enhanced after carbon ion beam combined with gemcitabine compared to carbon ion beam, X-ray alone or X-ray combined with gemcitabine, suggesting that carbon ion beam combined with chemotherapy may do more DNA damage to the cancer cells as a results activated homologous recombination pathway [51]. However, expressions of angiogenesis-related genes such as HIF1α, VEGF, CD34 and tumor invasion-related genes like MMP2, MMP9, E-cadherin and β-catenin were increased by either carbon ion beam or X-ray combined with gemcitabine compared to carbon ion beam or X-ray irradiation alone, implying that either high or low LET radiation may enhance tumor angiogenesis and/or invasion when combined with chemotherapy [52]. In accordance with previous reports [53], in the present study, the number of γH2AX foci formed in CSCs was significantly reduced after X-ray irradiation compared to that of non-CSCs, and not only the number but also the size of foci after carbon ion beam were significantly higher compared to that of X-ray irradiation, indicating that although CSC have more capacity to repair X-ray induced DSB, high LET carbon ion beam induced more complex DSB which is not easily repairable [40, 54, 55]. It has been reported that gemcitabine impairs cancer cells from repairing radiation-induced DNA damage by reducing the availability of normal nucleotides accompanied with elevation of residual γH2AX [46], and in this study, a larger number as well as larger-sized γH2AX foci formed when carbon ion beam combined with gemcitabine compared to that of X-rays alone, carbon ion beam alone or X-ray combined with gemcitabine. This finding could explain why a high LET carbon ion beam combined with chemotherapy has more potential to induce unrepairable complex cluster DSB. Taken together, our results are the first to show that predominant effects of carbon ion beam in combination with gemcitabine on pancreatic cancer cell killing mainly result from efficient eradication of CSCs rather than non-CSCs.

To determine the tumor growth control possibility by carbon ion beam in combination with gemcitabine, the xenograft tumors were also treated with carbon ion 25 Gy or X-ray 35 Gy alone or combined with 50 mg/kg gemcitabine ip. Carbon ion beam with 25 Gy in combination with gemcitabine can suppress tumor growth without re-growth after 7-8 weeks. This is consistent with our recent clinical trial with 58% of 2-year local control and 54% of 2-year overall survival by high LET carbon ion radiotherapy combined with gemcitabine, which is an almost 2 times better results compared to low LET photon radiotherapy combined with chemotherapy [6-8, 38]. As expected, treatment with 35 Gy X-ray in combination with gemcitabine failed to control tumor growth. Histopathological features showed that most of tumor cells did not disrupt by 30 Gy X-rays or 15 Gy carbon ion beam, but the tumor cells were partially destroyed by 25 Gy carbon ion beam. It is very clearly shown that most of the tumor cells were destroyed after irradiated with 35 Gy carbon-ion alone or 25 Gy carbon ion in combination with 50 mg/kg gemcitabine without significant side effects. The CD44 and ESA expression was slightly decreased with carbon ion beam at dose of 15 Gy, but significantly suppressed by either by 35 Gy of carbon ion beam alone or 25 Gy carbon ion beam combined with 50 mg/kg gemcitabine. In comparison, the expression of CD44 and ESA was increased by X-rays at a dose of 35 Gy, and still remained even combined with 50 mg/kg gemcitabine. This finding suggests that a high dose of carbon ion beam alone or relatively low dose of carbon ion beam combined with chemotherapy can effectively eradicate CSCs.

For the last several decades, conventional radiotherapy combined with chemotherapy has improved many cancer types such as bladder, gastric and rectal cancer treatment dramatically[56-58], and the mechanisms behind the synergistic effect of combing radiation and chemotherapy have been well documented [43, 59, 60]. During the past 20 years, carbon ion radiotherapy alone has been successful in treating many radiorsistant and recurrent refractory human cancers with a high local control rate, equal or better outcome compared to surgery and established minimally invasive, short-term and highest QOL treatment [23, 24, 26, 28, 30-32]. However, to not only further improve local control rate but also give systemic treatment to improve distant recurrence-free survival (DRFS) and thus disease-specific survival (DSS) for some advanced cancers for which it is difficult to elevate the radiation doses, combining chemotherapy with carbon ion beam is also highly desirable.

In summary, carbon ion beam combined with gemcitabine synergistically enhanced pancreatic CSCs death via inhibition of DNA repair as well as e irreparable complex DNA damage, increasing apoptosis and autophagy, and inhibition of cell proliferation at relatively low doses compared to carbon ion beams alone. Taken together, our findings show the potential benefits of carbon ion beams in combination with chemotherapy in targeting conventional radioresistant locally advanced pancreatic cancer.

## MATERIALS AND METHODS

### Cell lines and reagents

Human pancreatic cancer cell lines PK45, PNAC1, MIA PaCa-2 and BxPc-3 were purchased from American Type Culture Collection (Manassas, VA). Unsorted cells were cultured in Royal Park Memorial Institute(RPMI) supplemented with 10% heat-inactivated fetal calf serum (Beit-HaEmek, Israel),100 unit/mL penicillin and 100μg/mL streptomycin (Invitrogen) at 37°C with 5% CO2-in-air. The medium was changed every other day. CSCs and non-CSCs isolated from PK45, PNAC1, MIAPaCa-2 and BxPc-3 cells were cultured with serum-free Essential 8 medium (Life technologies Japan Ltd, Tokyo). Gemcitabine was purchased from Eli Lilly Japan. The gemcitabine solutions were diluted in PBS immediately before use.

### Colony and spheroid formation assays

Clonogenic survival assay was performed as described previously [39]. In brief, the appropriate plating density was aimed at producing 20–40 surviving colonies in each T-25 flask. After incubation for 14 days, the colonies were fixed and stained with 0.3% methylene blue in ethanol, and colonies containing more than 50 cells were counted as survivor. At least three parallel samples were scored in three to five repetitions performed for each irradiation condition. Clonogenicity and spheroid formation ability assays for CD44+/ESA+, CD44+/CD24+ and CD44−/ESA−, CD44−/CD24− cells sorted from PK45, PNAC1, MIAPaCa-2 and BxPc-3 cells plated in triplicate in a 6-cm dish or a 96 well spheroid formation plate (Sumilon, Sumitomo Bakelite Co., Ltd, Tokyo, Japan) were performed as described previously [40]. The data is presented as percentage of the wells that contained spheres. and the average size using WinRoof 5.6 software (Mitani Corporation, Tokyo, Japan) after 1-week incubation.

### Animals

NOD/SCID mice (6-8 weeks old, Charles River Laboratories, Yokohama, Japan) were maintained under defined conditions at the NIRS animal facility. The animals were observed for at least 12 weeks, and tumorigenicity was determined when tumor nodules were identified on their body surfaces. Tumor formation assays for CD44+/ESA+, CD44+/CD24+ and CD44−/ESA−, CD44−/CD24− cells were also performed as described previously [40]. For the xenograft tumor control study, NOD-SCID mice were subcutaneously injected with a 50 μl solution containing 1 × 10^6^ viable PK45 cells into the right thigh. *Mice* bearing 8-10 mm tumors were injected ip with gemcitabine (50 mg/kg) alone once a week (day 1, 8, 15, etc) or at times after a single fraction of carbon ion beam with 25 Gy or X-ray with 35 Gy for 3 weeks. All experiments involving the use of animals were performed in accordance with NIRS institutional animal welfare guidelines.

### Irradiation

Cells were irradiated with carbon-ion beams (accelerated by the HIMAC). Briefly, the initial energy of the carbon-ion beams was 290 MeV/n, center of 6 cm Spread-Out Bragg Peak (SOBP) with average LET 50 keV/μm. As a reference, cells were also irradiated with conventional 200 kVp X-ray (TITAN, GE Co., USA).

### FACS analysis

FACS analysis for the cells irradiated with X-rays or carbon ion beams was performed with *BD* FACS Aria (Becton Dickinson, San Jose, CA, USA) as described previously [39, 40]. In brief, the cells were prepared and labeled with conjugated anti-human CD44-PE (Miltenyi Biotec), ESA-APC (Miltenyi Biotec), and CD24-FITC. Isotype matched immunoglobulin served as control. Cells were incubated for 20 min at each step and were washed with 2% FCS/PBS between steps. The percentage of CD44+, ESA+, and CD24+ present was assessed after correction for the percentage of cells reactive with an isotype control.

### PCR profiler array analysis of various gene expressions

The Human Custom RT² Profiler™ PCR Array (CAPH11870A, Qiagen) profiles the expression of 42 genes involved in DNA damage, apoptosis, autophagy, senescence, stemness, and angiogenesis. RNA was purified using the Qiagen RNAeasy kit, including on-column DNAse treatment to remove genomic DNA. cDNA was prepared with the RT^2^ First Strand Kit (SABiosciences, Frederick, Maryland, USA). A PCR profiler array specific for 48 × 2 OSRGs was performed (RT^2^ SYBR Green/ROX qPCR Master Mix; SABiosciences) in 96-well microtiter plates on an ABI 7300 instrument (Applied Biosystems, California, USA). For data analysis, the ΔΔCt method was applied using the RT^2^ Profiler PCR Array software package was used and statistical analyses performed (n = 3). This package uses ΔΔ C_T_–based fold change calculations and the Student's *t*-test to calculate two-tail, equal variance p-values. The fold change from PK45 cells was calculated as 2^−ΔΔCt^. If the fold change was greater than 1, the result was considered as fold-upregulation. If the fold change was less than 1, the negative inverse of the result was considered as fold-downregulation [41].

### γH2AX immunofluorescence assay

Immunofluorescence staining of phospho-Histone H2AX (Ser139) (γH2AX) was performed as previously [40]. In brief, cultured cells grown on plastic chamber slides (Lab-Tek. Nunc, USA) were fixed in 4% formaldehyde, then permeabilized in 0.2% Triton X-100 and blocked with 10% goat serum, incubated with mouse monoclonal anti-γH2AX for at 37°C in PBS with 10% goat serum and washed with PBS. The cells were incubated with the Alexa 488 anti rabbit secondary antibody at 37°C in PBS with 10% goat serum and washed in PBS. Cover glasses were mounted in ProLong® Gold antifade reagent with DAPI (Invitrogen). Fluorescence images were captured using an Olympus DP70 fluorescence microscope for analysis. A minimum of 100 cells in each treatment group were counted. Nuclear γH2AX foci size was estimated by ImageJ 1.45 software (NIH).

### Gross morphology and histopathology

Gross morphological changes were followed up to 12 weeks after a single fraction of X-ray, carbon-ion beam alone or in combination with gemcitabine. At selected time points, tumors were excised and histopathological examinations were performed. Xenograft tumors from different groups were fixed in 10% neutral formalin and embedded in paraffin followed by sectioning (4 μm) onto slides. Sections were stained with hematoxylin and eosin (HE) and assessed microscopically.

### Immunofluorescence staining of cancer stem cell markers

The paraffin-embedded PK45 xenograft tumor sections were deparaffinization by xylene and rehydration by 100%, 95% ethanol and sections rinsed with dH2O. Then the slides were boiled in 10 mM sodium citrate buffer pH 6.0 for antigen unmasking. The block specimen was blocked in a blocking buffer (1X PBS/5% normal serum/0.3% Triton™ X-100) for 1h, after which diluted fluorochrome-conjugated primary antibody CD44-PE (BD Pharmingen™) and ESA-FITC (BD Pharmingen™) were applied and the specimen was incubated overnight at 4°C. After rinsing three times in 1X PBS for 5 min each, coversliped slides with Prolong® Gold Antifade Reagent (#9071) or Prolong® Gold Antifade Reagent with DAPI (#8961). Ten fields were selected and expression was evaluated in 10 fields with high power (x200) microscopy [39].

### Statistical analysis

One-way ANOVA and Bonferroni multiple comparison tests were used when mean differences between the groups were evaluated by StatView software (SAS Institute, Inc., Cary, NC). For all comparisons, *p* values less than 0.05 were defined as significant.

## References

[1] Schneider G, Siveke JT, Eckel F, Schmid RM (2005). Pancreatic cancer: basic and clinical aspects. Gastroenterology.

[2] Siegel R, Naishadham D, Jemal A (2012). Cancer statistics, 2012. CA: a cancer journal for clinicians.

[3] Werner J, Combs SE, Springfeld C, Hartwig W, Hackert T, Buchler MW (2013). Advanced-stage pancreatic cancer: therapy options. Nature reviews Clinical oncology.

[4] Ben-Josef E, Lawrence TS (2012). Radiotherapy: the importance of local control in pancreatic cancer. Nature reviews Clinical oncology.

[5] Bardeesy N, DePinho RA (2002). Pancreatic cancer biology and genetics. Nature reviews Cancer.

[6] Schellenberg D, Kim J, Christman-Skieller C, Chun CL, Columbo LA, Ford JM, Fisher GA, Kunz PL, Van Dam J, Quon A, Desser TS, Norton J, Hsu A, Maxim PG, Xing L, Goodman KA (2011). Single-fraction stereotactic body radiation therapy and sequential gemcitabine for the treatment of locally advanced pancreatic cancer. International journal of radiation oncology, biology, physics.

[7] Sudo K, Yamaguchi T, Ishihara T, Nakamura K, Hara T, Denda T, Tawada K, Imagumbai T, Araki H, Sakai M, Hatano K, Kawakami H, Uno T, Ito H, Yokosuka O (2011). Phase II study of oral S-1 and concurrent radiotherapy in patients with unresectable locally advanced pancreatic cancer. International journal of radiation oncology, biology, physics.

[8] Small W, Mulcahy MF, Rademaker A, Bentrem DJ, Benson AB, Weitner BB, Talamonti MS (2011). Phase II trial of full-dose gemcitabine and bevacizumab in combination with attenuated three-dimensional conformal radiotherapy in patients with localized pancreatic cancer. International journal of radiation oncology, biology, physics.

[9] Burris HA, Moore MJ, Andersen J, Green MR, Rothenberg ML, Madiano MR, Cripps MC, Portenoy RK, Storniolo AM, Tarassoff P, Nelson R, Dorr FA, Stephens CD, VanHoff DD (1997). Improvements in survival and clinical benefit with gemcitabine as first-line therapy for patients with advanced pancreas cancer: A randomized trial. Journal of Clinical Oncology.

[10] Li C, Heidt DG, Dalerba P, Burant CF, Zhang L, Adsay V, Wicha M, Clarke MF, Simeone DM (2007). Identification of pancreatic cancer stem cells. Cancer research.

[11] Hermann PC, Huber SL, Herrler T, Aicher A, Ellwart JW, Guba M, Bruns CJ, Heeschen C (2007). Distinct populations of cancer stem cells determine tumor growth and metastatic activity in human pancreatic cancer. Cell stem cell.

[12] Meacham CE, Morrison SJ (2013). Tumour heterogeneity and cancer cell plasticity. Nature.

[13] Hatina J (2012). The dynamics of cancer stem cells. Neoplasma.

[14] Monteiro J, Fodde R (2010). Cancer stemness and metastasis: therapeutic consequences and perspectives. European journal of cancer (Oxford, England : 1990).

[15] Rich JN (2007). Cancer stem cells in radiation resistance. Cancer research.

[16] Dingli D, Michor F (2006). Successful therapy must eradicate cancer stem cells. Stem cells.

[17] Ning X, Shu J, Du Y, Ben Q, Li Z (2013). Therapeutic strategies targeting cancer stem cells. Cancer biology & therapy.

[18] Penchev VR, Rasheed ZA, Maitra A, Matsui W (2012). Heterogeneity and targeting of pancreatic cancer stem cells. Clinical cancer research : an official journal of the American Association for Cancer Research.

[19] Chen K, Huang YH, Chen JL (2013). Understanding and targeting cancer stem cells: therapeutic implications and challenges. Acta pharmacologica Sinica.

[20] Hambardzumyan D, Squartro M, Holland EC (2006). Radiation resistance and stem-like cells in brain tumors. Cancer Cell.

[21] Pignalosa D, Durante M (2012). Overcoming resistance of cancer stem cells. The Lancet Oncology.

[22] Vermeulen L, de Sousa e Melo F, Richel DJ, Medema JP (2012). The developing cancer stem-cell model: clinical challenges and opportunities. The Lancet Oncology.

[23] Tsujii H, Kamada T (2012). A review of update clinical results of carbon ion radiotherapy. Japanese journal of clinical oncology.

[24] Mizoe JE, Hasegawa A, Jingu K, Takagi R, Bessyo H, Morikawa T, Tonoki M, Tsuji H, Kamada T, Tsujii H, Okamoto Y, Organizing Committee for the Working Group for Head Neck C (2012). Results of carbon ion radiotherapy for head and neck cancer. Radiotherapy and oncology : journal of the European Society for Therapeutic Radiology and Oncology.

[25] Matsumoto K, Imai R, Kamada T, Maruyama K, Tsuji H, Tsujii H, Shioyama Y, Honda H, Isu K (2013). Impact of carbon ion radiotherapy for primary spinal sarcoma. Cancer.

[26] Imai R, Kamada T, Tsuji H, Sugawara S, Serizawa I, Tsujii H, Tatezaki S, Working Group for B and Soft Tissue S (2010). Effect of carbon ion radiotherapy for sacral chordoma: results of Phase I-II and Phase II clinical trials. International journal of radiation oncology, biology, physics.

[27] Nishida Y, Kamada T, Imai R, Tsukushi S, Yamada Y, Sugiura H, Shido Y, Wasa J, Ishiguro N (2011). Clinical outcome of sacral chordoma with carbon ion radiotherapy compared with surgery. International journal of radiation oncology, biology, physics.

[28] Toyama S, Tsuji H, Mizoguchi N, Nomiya T, Kamada T, Tokumaru S, Mizota A, Ohnishi Y, Tsujii H, Working Group for Ophthalmologic T (2013). Long-term results of carbon ion radiation therapy for locally advanced or unfavorably located choroidal melanoma: usefulness of CT-based 2-port orthogonal therapy for reducing the incidence of neovascular glaucoma. International journal of radiation oncology, biology, physics.

[29] Yanagi T, Mizoe JE, Hasegawa A, Takagi R, Bessho H, Onda T, Kamada T, Okamoto Y, Tsujii H (2009). Mucosal malignant melanoma of the head and neck treated by carbon ion radiotherapy. International journal of radiation oncology, biology, physics.

[30] Imai R, Kamada T, Tsuji H, Yanagi T, Baba M, Miyamoto T, Kato S, Kandatsu S, Mizoe JE, Tsujii H, Tatezaki S, Working Group for Bone STS (2004). Carbon ion radiotherapy for unresectable sacral chordomas. Clinical cancer research : an official journal of the American Association for Cancer Research.

[31] Loeffler JS, Durante M (2013). Charged particle therapy--optimization, challenges and future directions. Nature reviews Clinical oncology.

[32] Durante M, Loeffler JS (2010). Charged particles in radiation oncology. Nature reviews Clinical oncology.

[33] Okayasu R (2012). Repair of DNA damage induced by accelerated heavy ions--a mini review. International journal of cancer Journal international du cancer.

[34] Combs SE, Habermehl D, Kieser M, Dreher C, Werner J, Haselmann R, Jakel O, Jager D, Buchler MW, Debus J (2013). Phase I study evaluating the treatment of patients with locally advanced pancreatic cancer with carbon ion radiotherapy: the PHOENIX-01 trial. BMC cancer.

[35] Shinoto M, Yamada S, Yasuda S, Imada H, Shioyama Y, Honda H, Kamada T, Tsujii H, Saisho H, Working Group for Pancreas C (2013). Phase 1 trial of preoperative, short-course carbon-ion radiotherapy for patients with resectable pancreatic cancer. Cancer.

[36] Schlaich F, Brons S, Haberer T, Debus J, Combs SE, Weber KJ (2013). Comparison of the effects of photon versus carbon ion irradiation when combined with chemotherapy *in vitro*. Radiation oncology.

[37] Schlaich F, Brons S, Haberer T, Debus J, Combs SE, Weber KJ (2013). Comparison of the effects of photon versus carbon ion irradiation when combined with chemotherapy *in vitro*. Radiation oncology.

[38] Yamada S, Terashima K, Shinoto M, Yasuda S (2014). Pancreatic Cancer. Carbon-Ion Radiotherapy Principles, Practices, and Treatment.

[39] Cui X, Oonishi K, Tsujii H, Yasuda T, Matsumoto Y, Furusawa Y, Akashi M, Kamada T, Okayasu R (2011). Effects of carbon ion beam on putative colon cancer stem cells and its comparison with X-rays. Cancer research.

[40] Oonishi K, Cui X, Hirakawa H, Fujimori A, Kamijo T, Yamada S, Yokosuka O, Kamada T (2012). Different effects of carbon ion beams and X-rays on clonogenic survival and DNA repair in human pancreatic cancer stem-like cells. Radiotherapy and oncology : journal of the European Society for Therapeutic Radiology and Oncology.

[41] Steg AD, Bevis KS, Katre AA, Ziebarth A, Dobbin ZC, Alvarez RD, Zhang K, Conner M, Landen CN (2012). Stem cell pathways contribute to clinical chemoresistance in ovarian cancer. Clinical cancer research : an official journal of the American Association for Cancer Research.

[42] Suzuki M, Kase Y, Kanai T, Ando K (2000). Change in radiosensitivity with fractionated-dose irradiation of carbon-ion beams in five different human cell lines. International journal of radiation oncology, biology, physics.

[43] Stewart JM, Shaw PA, Gedye C, Bernardini MQ, Neel BG, Ailles LE (2011). Phenotypic heterogeneity and instability of human ovarian tumor-initiating cells. Proc Natl Acad Sci U S A.

[44] Mukubou H, Tsujimura T, Sasaki R, Ku Y (2010). The role of autophagy in the treatment of pancreatic cancer with gemcitabine and ionizing radiation. International journal of oncology.

[45] Pauwels B, Vermorken JB, Wouters A, Ides J, Van Laere S, Lambrechts HA, Pattyn GG, Vermeulen K, Meijnders P, Lardon F (2009). The role of apoptotic cell death in the radiosensitising effect of gemcitabine. British journal of cancer.

[46] Morgan MA, Meirovitz A, Davis MA, Kollar LE, Hassan MC, Lawrence TS (2008). Radiotherapy Combined with Gemcitabine and Oxaliplatin in Pancreatic Cancer Cells. Translational Oncology.

[47] Kurenova E, Liao J, He DH, Hunt D, Yemma M, Bshara W, Seshadri M, Cance WG (2013). The FAK scaffold inhibitor C4 disrupts FAK-VEGFR-3 signaling and inhibits pancreatic cancer growth. Oncotarget.

[48] Modrak DE, Leon E, Goldenberg DM, Gold DV (2009). Ceramide regulates gemcitabine-induced senescence and apoptosis in human pancreatic cancer cell lines. Mol Cancer Res.

[49] vHerreros-Villanueva M, Bujanda L, Billadeau DD, Zhang JS (2014). Embryonic stem cell factors and pancreatic cancer. World journal of gastroenterology : WJG.

[50] Ghisolfi L, Keates AC, Hu X, Lee DK, Li CJ (2012). Ionizing radiation induces stemness in cancer cells. PloS one.

[51] Zafar F, Seidler SB, Kronenberg A, Schild D, Wiese C (2010). Homologous recombination contributes to the repair of DNA double-strand breaks induced by high-energy iron ions. Radiation research.

[52] Kuonen F, Secondini C, Ruegg C (2012). Molecular pathways: emerging pathways mediating growth, invasion, and metastasis of tumors progressing in an irradiated microenvironment. Clinical cancer research : an official journal of the American Association for Cancer Research.

[53] Al-Assar O, Muschel RJ, Mantoni TS, McKenna WG, Brunner TB (2009). Radiation response of cancer stem-like cells from established human cell lines after sorting for surface markers. International journal of radiation oncology, biology, physics.

[54] Yajima H, Fujisawa H, Nakajima NI, Hirakawa H, Jeggo PA, Okayasu R, Fujimori A (2013). The complexity of DNA double strand breaks is a critical factor enhancing end-resection. DNA repair.

[55] Nakajima NI, Brunton H, Watanabe R, Shrikhande A, Hirayama R, Matsufuji N, Fujimori A, Murakami T, Okayasu R, Jeggo P, Shibata A (2013). Visualisation of gammaH2AX foci caused by heavy ion particle traversal; distinction between core track versus non-track damage. PloS one.

[56] Plataniotis GA, Dale RG (2014). Assessment of the radiation-equivalent of chemotherapy contributions in 1-phase radio-chemotherapy treatment of muscle-invasive bladder cancer. International journal of radiation oncology, biology, physics.

[57] Collette L, Bosset JF, den Dulk M, Nguyen F, Mineur L, Maingon P, Radosevic-Jelic L, Pierart M, Calais G, European Organisation for R, Treatment of Cancer Radiation Oncology G (2007). Patients with curative resection of cT3-4 rectal cancer after preoperative radiotherapy or radiochemotherapy: does anybody benefit from adjuvant fluorouracil-based chemotherapy? A trial of the European Organisation for Research and Treatment of Cancer Radiation Oncology Group. Journal of clinical oncology : official journal of the American Society of Clinical Oncology.

[58] Smalley SR, Benedetti JK, Haller DG, Hundahl SA, Estes NC, Ajani JA, Gunderson LL, Goldman B, Martenson JA, Jessup JM, Stemmermann GN, Blanke CD, Macdonald JS (2012). Updated analysis of SWOG-directed intergroup study 0116: a phase III trial of adjuvant radiochemotherapy versus observation after curative gastric cancer resection. Journal of clinical oncology : official journal of the American Society of Clinical Oncology.

[59] Steel GG (1988). The search for therapeutic gain in the combination of radiotherapy and chemotherapy. Radiotherapy and oncology : journal of the European Society for Therapeutic Radiology and Oncology.

[60] Mamon HJ, Tepper JE (2014). Combination Chemoradiation Therapy: The Whole Is More Than the Sum of the Parts. Journal of Clinical Oncology.

